# Present sample identification systems

**DOI:** 10.1155/S1463924681000552

**Published:** 1981

**Authors:** H. Keller

**Affiliations:** Institut fuer Klinische Chemic und Haematologie St. Gallen Switzerland


### Present sample identification systems*

**H. Keller**

*Institut fuer Klinische Chemie und Haematologie, St. Gallen, Switzerland.*

The theme "Sample identification" was last discussed at the international meeting "Biochemische Analytik 78". At that time it was stated that publications dealing with sample identification are relatively few and in recent years there have been no important innovations in this field. This statement is valid today with one exception: in the new edition of the well-known book by R. Haeckel "Rationalisierung des Medizinischen Laboratoriums" [1] there is a very competent contribution from Dres. Mieth und Porth, dealing with the techniques of sample identification.

The term "sample identification" is a paraphrase for all instructions, equipment and processes for the recognition and allocation of a specimen or sample, and an analytical result to a distinct source of specimen.

The importance of sample identification depends on:

1. The number of different specimens to be analysed in a given period of time.
2. The number of different tests routinely practiced by the laboratory.
3. The kind of equipment being used.
4. The standard of organisation.
5. The distance between laboratory and patient.

The influence of these five points has become particularly evident since computers were introduced into clinical laboratories. The increasing volume of samples and tests and the distance between the patient and the laboratory are important factors. More important as an aid to sample identification is the proper selection of analysers and data processors, linked to good organisation. Bad organisation and a random collection of equipment aggravate these problems immensely.

There are three essential components to sample identification:

1. One fundamental requirement is a complete identification statement, inseparably linked to the specimen.
2. The mechanism of intralaboratory identification consists of generating an identification symbol fixed to the sample during all processes of transport and transfer.
3. The goal is the permanent control of identification during the analytical processes, calculations of results, and presentation of reports.

The complete identification statement consists of information about source of specimen and request, kind of material, ordered tests and instructions, and finally the timing.

Each sample transfer step holds a trap. The first one is when a sample is taken from a patient. The next transfer takes place in the laboratory when the sample is split and distributed to process tubes. Here, the identification is taken and transferred to the tubes in a coded form. Every repetition of this process increases the probability of loss of data or wrong allocation.

Sample identification begins with the identification of the patient. His name and/or a relevant symbol (eg an I-number) must be taken and linked to the specimen. In order to increase the safety of this important step, some recommendations are made. The system proposed by Rubin [2] seems to be very effective but nevertheless it is not offered by laboratory suppliers. Adhesive labels are generally used; these are preprinted by the hospitals at admission or are reprints from identification-cards. Either the same labels are used for both the request-card and the container, or the specimen container has its own symbol for identification.

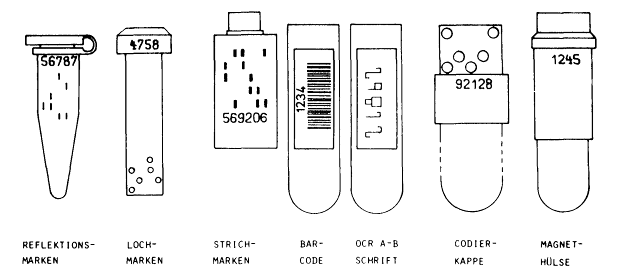

*Figure 1. Methods of direct sample identification*

Within the lab a system or an organisation for all processes of identification has to be installed and four sub-types can be distinguished which are either indirect or direct methods. Indirect sample identification is an old-fashioned method, which should really be eliminated. It has two variants:

1. In *positional identification*, the sample is identified from its position in a chain of samples, a magazine, or sample plate etc which, in turn, is related to a previously created list. Identification may take place, for example, by counting the members of the chain, whereby regular empty spaces act as markers.

2. If only the order of samples, together with a work list, is used for identification, then the procedure is called *serial identification*. This latter method carries the greatest danger of sample interchange in the laboratory.

3. *Direct sample identification without splitting* is based on the concept of the so-called consecutive distribution [3], first realised by Roland Richterich on his Greiner-GSA [4]. The specimen container is put directly into the analyser (after centrifugation). The card for identification and request serves as carrier of the results. A similar mode is possible on big modern analysers, and also on dedicated systems, eg for blood glucose. This is the cheapest form of sample identification and is also the safest.

4. *Direct sample identification with splitting* requires more operator time. In routine operation, the complete identity statement is usually too extensive and contains more information than is required for the analytical process. For this reason, the statement is abbreviated, with the aid of a code, to an identification symbol. This may consist of a patient number, day number and/or other relevant numbers, eg the nature of the test or tests required. Specimen containers, process vessels, and analytical records are linked to one another by the identification symbols. Retrieval of the complete identification statement necessitates a decoding process, which may be based on, for example a "day list". Most simply, a manually prepared list is used, while in

---

**Paper based on a meeting of a working group of German Society of Clinical Chemistry, held in Munich, April 26–27, 1980.*

*This the second in a series of three papers. The third will appear in a future issue.*

better equipped laboratories, these lists are stored in an electronic data processor.

For tube marking, some commercial systems have been developed (Figure 1). The Eppendorf labelling system is based on reflection marks imprinted on the process vessels. The marks, which are read automatically by the machine, represent patient or day numbers, and correspond to a five channel binary code. They are supplemented by visual characters so that an operator check is possible at any time. This particular identification system is widely used by laboratories in central Europe [5].

The SILAB system is similar, but it employs a different labelling technique. The system has been discontinued however [6].

Label-reading by machine was used by Technicon in the IDEE-system and it has been retained in a modified form. This method is especially widely used in Great Britain, but it is also popular in European laboratories [7].

Forerunner of this system was the IBM 1080 data system with perforated punched cards which was introduced by Rappoport [8] in the USA, and which is still in use in many American and a few European laboratories. There have been many interesting modifications of the perforated punched card, eg by Borner [9], by Jentzsch [10] and others.

Renewed interest is being shown in the Bar-code technique [11,12,13]. Identification labels are not restricted to any one kind of vessel; in addition to specimen tubes, they can also be used for haematocrit capillaries, urine vessels, blood sugar tubes etc.

Labels and analysis requests carry the bar-code which can also be read visually (Figure 2). The success of this system is due to the fact that the reading process is especially simple; it can be performed rapidly and reliably, either manually with the aid of a reading pistol, or mechanically with a laser beam. It should be pointed out that the reading unit, which is required in order to achieve high reading reliability, is relatively cheap. With the aid of the EDP, bar-code labels are produced for primary serum vessels and for all the other process vessels. The labels carry the identification number of the patient, together with the investigation to be performed. Experience has shown that reading reliability is not endangered even by badly smudged labels.

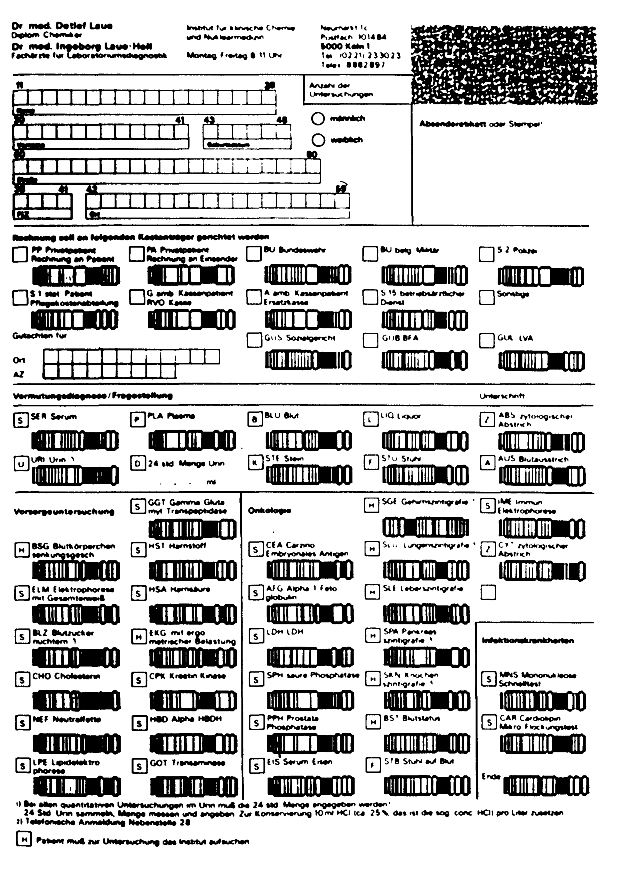

*Figure 2. Example of the use of Bar-code request sheets (with the permission of Dr Laue, Cologne)*

The most recent and perhaps most promising development is optical character recognition (OCR). Optical character sets for OCR at present in common use for single scanners are shown in Figure 3. Provisionally, it is possible to read every digit, although a more economically priced reading apparatus will limit the reading ability to the first ten letters. Operation of the reading pistol, such as the Eppendorf ELIS system, is extremely simple; it is error-free and relatively quick to use even for non-specialised personnel [14].

OCR offers the following advantages:

1. It is a direct code, and the information can be read both automatically and visually.
2. No special printing apparatus is needed; the labels and other information carrier can be prepared with any golf-ball typewriter, or EDP-controlled rapid printer.
3. In the foreseeable future, OCR systems will be able to read automatically the name of the patient and other alpha-numerical information.
4. OCR labelling and reading are becoming increasingly popular in the commerical field. Development should therefore be accelerated, and the prices of the reading apparatus should come down.
5. The adaptation of OCR-readers to the common analysers should be as easy as for bar-code readers.

Disadvantages of the OCR-reader at the moment are the limited number of symbols and the relatively slow manual operation of the reading pistol. Other concepts such as a coded cap by Silab, a magnetic collar by Marksteiner, or magnetic caps fitted to the Hycel Marc X found no acceptance [15].

From the foregoing discussion, it is clear that in all methods presently employed, it is not the material under investigation that is identified with respect to the source of the material but the sample container and/or chain of containers. The source of material and the corresponding analytical results can only be related by a merging process.

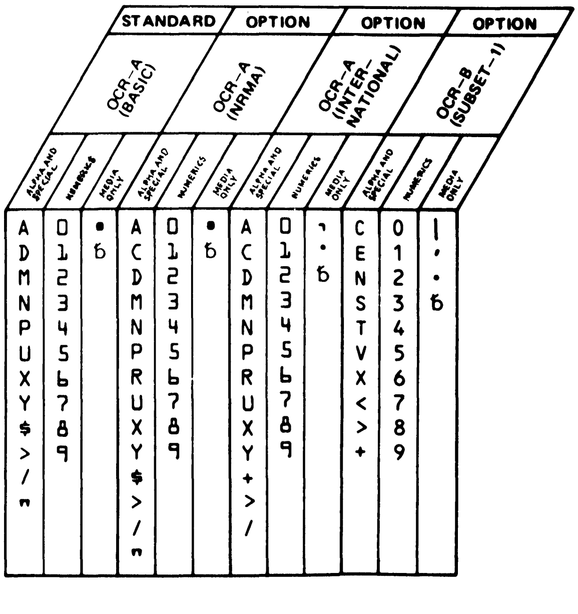

*Figure 3. Optional character sets of OCR*

Nevertheless there are two fundamentally different ways of identifying sets of data. First, the cumulative collection of test results may be checked for plausibility. Mislabelled results will be recognised if they differ greatly from those recorded earlier. The method, however, is not very effective as Sheiner *et al* [16] have shown. A Delta check method, controlled by a computer, discovered only 50% of mislabelled specimens. On the other hand only 10% of the data suspected of being mislabelled were in fact wrongly identified.

The second approach is based on the biochemical individuality characterising a healthy, or a sick person unequivocally, if a sufficient number of tests is performed. Young and his co-workers [17] have recently demonstrated that the identity is reflected by multivariate data of biochemical test profiles. Profiles of individual samples were graphically displayed as computer-drawn faces and they conclude from their experience the following: "Discriminant functions can be used to detect mislabelled specimens in research studies and potentially in the clinical laboratory to detect misidentified samples from patients". Obviously this will not be a practicable method for sample identification in our laboratories. But reflecting on the coherence of individuality and identification may be worth while.

REFERENCES

[1] Haeckel, R. (1980), Rationalisierung des medizinischen Laboratoriums GIT Verlag Darmstadt 2nd. Ed.
[2] Rubin, M. (1969), VII Int Congr Clin Chem, Geneva Diskussionsbeitrag.
[3] Keller, H. and Richterich, R. (1972), VIII World Congress of Anatomic and Clinical Pathology Munich 12–16 Sept, Excerpta Medica Amsterdam Internat Congr Series No 285, 183-189.
[4] Richterich, R. and Greiner, R. (1971), *Z Klin Chem Klin Biochem,* 9, 187-193.
[5] Hille, W. and Knaus, M. (1973), *GIT Fachz Lab,* **17**, 1255.
[6] Knedel, M. (1971), Siemens Druckschrift Nr. MC 50/1016.
[7] Sher, P. P. and Gambino, S. R. (1970), *Adv Autom Analysis,* **1**, 63-65, Thurmann Ass Miami, Florida.
[8] Rappoport, A. E., Gennaro, W. D., and Constandse, W. J. (1968), *Modern Hospital,* **110**, 100-103.
[9] Borner, K. and Klein, E. (1969), *Z Klin Chem Klin Biochem,* 7, 185-188.
[10] Jentzsch, D. (1969), Probenidentifizierung bei klinisch-chemischen Analysen. Berichte zur klinischen Chemie. Perkin Elmer & Co. Ueberlingen/Bodensee Heft 1.
[11] Dudek, J., Roka, L. and Michel, H. (1974), *Aerztl Lab,* **20**, 430-436.
[12] Laue, D. (1977), The Bar-code: A universal system of identification in the medical laboratory. Birmingham 2nd Internat. Conference, Sept 12–16.
[13] American Blood Commission (1976), CCBBA special issue: August 1976, New blood pack label being tested. Am Blood Commission, Arlington, Va 22209.
[14] Michel, H. (1977), *Biotechn Umschau,* **1**, 23.
[15] Reviewed by Keller, H. (1979), *J Clin Chem Clin Biochem,* **17**, 57-64.
[16] Sheiner, L. B., Wheeler, L. A. and Moore J. K. (1979), *Clin Chem,* **25**, 2034-2037.
[17] Robertson, A. E., Steirteghem, A. C., Byrkit, J. E. and Young, D. S. (1980), *Clin Chem,* **26**, 30-36.

### An automated titration system

**T. Pap, M. Molnár and J. Inczédy**

*Institute of Analytical Chemistry, University of Veszprem, Hungary*

### Introduction

The automation of titrations has a long history, but since the introduction of digital computers into laboratories interest in and the possibilities of automation have significantly increased. Johansson [1,2] developed software packages for the calculation of concentration and equilibrium constant data from acid-base titration curves. Leggett [3] connected an automatic burette to a microprocessor and controlled the addition of the titrant to the solution. Similar systems were developed by Firstenberg [4], Betteridge *et al* [5], Nowogrocki [6] and recently Gampp, Maeder, Zuherbükler and Kaden [7].

This system is used for the titration of acid mixtures. The protonation constants of the conjugated bases were determined by additional titration of the solution of the single acids.

In the course of the work reported here, the automatic titration system was constructed from commercially available units. The system could be used to carry out titrations of mixtures and to calculate equivalency points, equilibrium constants and to print out results and record the titration curves.

### Titration system

The block scheme of the titration system can be seen in Figure 1. The control unit is a desk top calculator (EMG 666, Hungary) of 8 KB capacity. It is connected to a mosaic printer (EMG 896, Hungary), a plotter (Videoton NE-2000-666, Hungary), an impulse burette (Metrohm 111, Switzerland) and a digital voltmeter (MIKI, Type 1747, Hungary). A precision pH-meter (Radelkis OP 208, Hungary) and a combined glass-calomel electrode (Radelkis OP-8083, Hungary) were used for the acid-base titrations. The analog signal of the pH-meter was fed to the digital voltmeter and the digital signal of the voltmeter was transferred to the calculator via an interface (MIKI, Type 2702, Hungary). Another interface (EMG 79845, Hungary) was used to connect the impulse burette and the calculator.

The operation of the system for acid-base titrations is as follows. During the titration the calculator calculates the volume of the next titrant fraction from the value of the pH change using a given mathematical function and gives the command to the burette for a proper number of impulses. 0.001 ml titrant is delivered at each impulse. After the addition of the titrant, the solution is allowed to equilibrate and a command is given to store the measured pH value. The titration is continued until the burette is empty (10 ml). Finally, the evaluation process starts.

As mentioned earlier, there is an interface unit between the calculator and the burette which can be seen in Figure 2. The original unit was extended with a home-made coupling board to facilitate the above mentioned operations. This

